# Domestic Solid Fuel Use and Respiratory Health: Implications for Climate Change

**DOI:** 10.1093/geroni/igaf122.793

**Published:** 2025-12-31

**Authors:** Shaun Scholes, Laura Horsfall, Paola Zaninotto

**Affiliations:** University College London, London, England, United Kingdom; University College London, London, England, United Kingdom; University College London, London, England, United Kingdom

## Abstract

The domestic use of solid fuels such as coal and wood for heating is a significant source of air pollution, contributing to poor indoor and outdoor air quality and exacerbating climate change through the release of particulate matter and black carbon. These emissions not only accelerate global warming but also pose serious risks to human health, particularly for older adults who are more vulnerable to respiratory diseases. Despite growing concerns about air pollution and climate change, longitudinal evidence on the health impacts of domestic solid fuel use remains limited. This study explores how the use of solid fuels affects lung function trajectories among older adults, using FEV1 data from the English Longitudinal Study of Ageing (ELSA) from 2004-05 to 2012-13. Preliminary findings indicate that domestic solid fuel use was most common in both affluent households (20% in the highest wealth tertile) and fuel-poor households (19%) at wave 2. Over the 8-year study period, lung function declined more rapidly among those using solid fuels. For participants aged 70-79, FEV1 decreased on average by 0.12 litres among solid fuel users, compared to 0.07 litres among non-users. These results highlight the urgent need for public health and environmental policies promoting the transition to cleaner, more sustainable domestic energy sources. Given the dual burden of air pollution on both human health and climate change, reducing reliance on solid fuels is essential to protect older adults from respiratory decline while mitigating the broader environmental impacts of household emissions.

